# Supplementary material for: Weight-four parity checks in a spin-shuttling architecture
Source: Nature. 2026 Jul 29;655(8125):1160–6. doi: 10.1038/s41586-026-10766-3 (PMC13421349; doi:10.1038/s41586-026-10766-3)
Supplement: Supplementary file 1 — Supplementary Notes 1–3, Figs. 1–11 and Table 1. [file 41586_2026_10766_MOESM1_ESM.pdf]

nature portfolio

https://doi.org/10.1038/s41586-026-10766-3

Supplementary information

Weight-four parity checks in a spin-shuttling architecture

In the format provided by the authors and unedited

Supplementary Information: Weight-four parity checks in a spin-shuttling architecture

Brennan Undseth,[1, *] Nicola Meggiato,[1, *] Yi-Hsien Wu,[1] Sam R. Katiraee-Far,[1] Larysa Tryputen,[2] Sander L. de Snoo,[1] Davide Degli Esposti,[1] Giordano Scappucci,[1] Eliška Greplová,[1] and Lieven M. K. Vandersypen[1, †]

[1] *QuTech and Kavli Institute of Nanoscience, Delft University of Technology, Lorentzweg 1, 2628 CJ Delft, The Netherlands*
[2] *Netherlands Organization for Applied Scientific Research (TNO), Stieltjesweg 1, 2628 CK Delft, The Netherlands*

This supplementary information includes:

- Supplementary Note 1: Electrostatic Design
  - Supplementary Figure 1: Electrostatic Design
- Supplementary Note 2: Magnetostatic Design
  - Supplementary Figure 2: Magnetostatic Design
- Supplementary Note 3: Towards logical Loss-DiVincenzo spin qubits
  - Supplementary Figure 3: $[\![4, 2, 2]\!]$ code implementation
- Supplementary Figures 4-11
  - Supplementary Figure 4: Shuttling bus IV characteristics
  - Supplementary Figure 5: Bus stop DC
  - Supplementary Figure 6: Single-spin remote tuning
  - Supplementary Figure 7: Spin reloading
  - Supplementary Figure 8: Resonant gate calibration
  - Supplementary Figure 9: Phase crosstalk calibration
  - Supplementary Figure 10: DCZ calibration
  - Supplementary Figure 11: Residual exchange measurements
- Supplementary Table I: Character randomized benchmarking

* These authors contributed equally to this work.

† Corresponding author: l.m.k.vandersypen@tudelft.nl

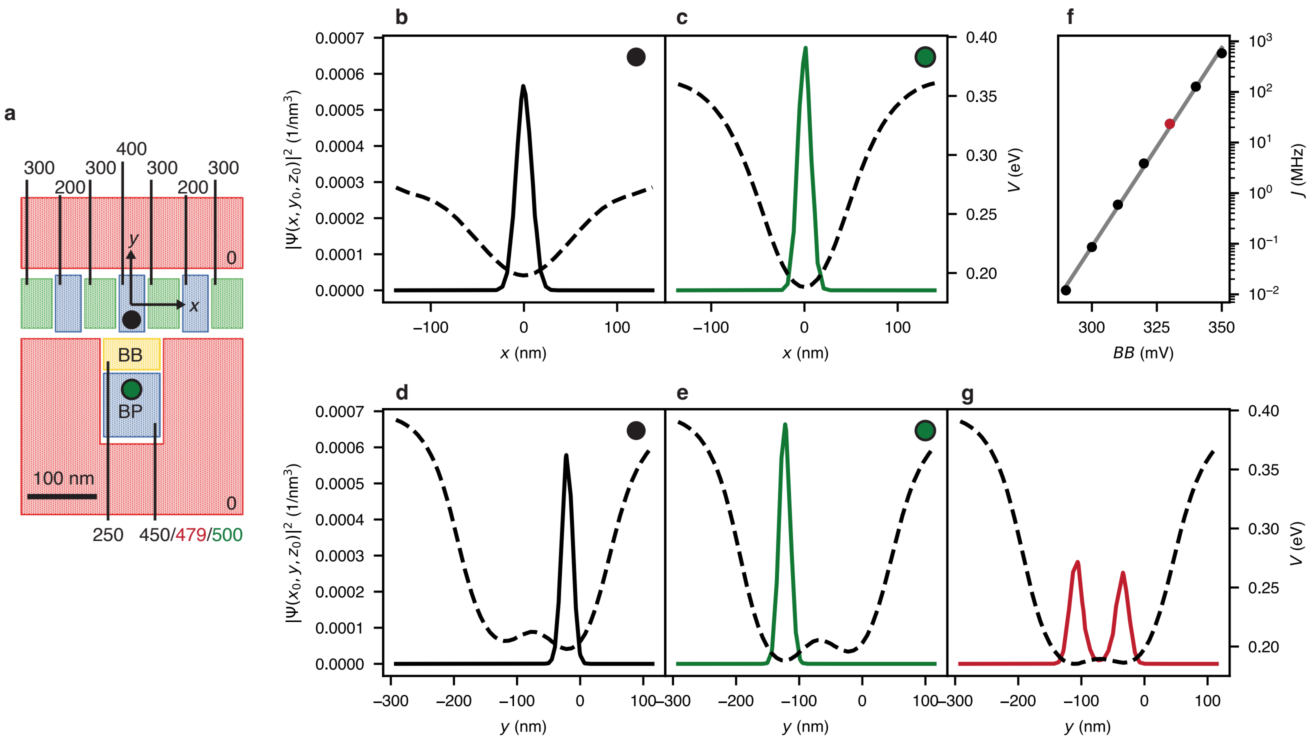

Supplementary Figure 1. **a** Illustration of the boundary conditions used for electrostatic simulations of a double-dot potential at one of the bus stops. All numeric labels indicate a Dirichlet boundary condition in millivolts for the Poisson solver, which are set to approximate the potential landscape when a travelling-wave potential minimum is adjacent to the bus stop. Depending on the voltage applied to the plunger BP, the potential minimum is localized in the shuttling bus (black) or bus stop (green). **b** x-axis linecut of the potential and spatial wavefunction (probability density) of the ground state when the potential minimum is in the shuttling bus. **c** x-axis linecut of the potential and spatial wavefunction of the ground state when the potential minimum is in the bus stop. **d** y-axis linecut of the potential and spatial wavefunction of the ground state when the potential minimum is in the shuttling bus. **e** y-axis linecut of the potential and spatial wavefunction of the ground state when the potential minimum is in the bus stop. **f** Estimated relationship between barrier voltage BB and exchange coupling as calculated from the perturbative relationship $J = 4t_c^2/U$ where the onsite Coulomb potential $U \approx 12\,\text{meV}$ for all voltage settings. The linear fit corresponds to $J = J_{\text{off}} \exp(0.18BB/\text{mV})$. **g** y-axis linecut of the potential and spatial wavefunction of the ground state when the double-dot potential is balanced at the voltage setting highlighted in **f**. The orbital energy spacing to the first excited state is equal to $2t_c$.

Supplementary Note 1. Electrostatic design

We use QTCAD to perform Poisson and Schrödinger simulations on a simplified device model [1]. We treat these simulations as a qualitative confirmation of intuition rather than an attempt at rigorous device modeling. We use a subset of the nominal gate design for a bus stop as shown in Supplementary Fig. 1a and perform a 3D Poisson simulation of the electrostatics using the indicated gate voltages as Dirichlet boundary conditions. These voltages are physically reasonable but generally smaller than those used in experiment due to screening effects which are not considered here. The bounding area for the simulation is kept large enough such that the natural boundary conditions do not affect the converged solution. The electrostatic solution is used as the static potential for a 3D time-independent Schrödinger equation solver to find the eigenspectrum of a single-electron in the effective double-dot potential.

Supplementary Fig. 1b-e illustrates x- and y-axis linecuts of the ground state when a single-electron is confined in the effective double-dot potential formed when the traveling-wave potential minimum is positioned adjacent to the bus stop. The simulated potential minima shift orthogonally to the axis of the shuttling bus as a result of the presence of the bus stops, as illustrated in Supplementary Fig. 2a. Depending on the bus stop plunger voltage BP, the electron's orbital ground state is either localized in the shuttling bus or the bus stop. We can therefore extract estimated positions of the spin qubits as input to magnetostatic simulations as detailed in Sec. Supplementary Note

2. Furthermore, we extract orbital energy splittings of 3.8 meV for the shuttled dot and 4.1 meV for the bus stop dot which are relevant for EDSR. Using a simplified many-body simulation, the onsite Coulomb potential is estimated to be 12 meV for both dots.

The microscopic valley degree of freedom is not accounted for in the simulations. Nevertheless, the tunability of the bare tunnel coupling t_c may be extracted from the energy difference $2t_c$ between bonding and antibonding states when the double-dot potential is balanced. An example is shown in Supplementary Fig. 1g. The bare exchange coupling follows from perturbation theory as $J = 4t_c^2/U$ [2]. The true exchange coupling in silicon will be influenced by the valley phase difference between the occupied eigenstate of each dot. However, $J \propto t_c^2$ remains true. Therefore, the barrier lever arm can be estimated by fitting to a model $J = J_{\text{off}} \exp(\alpha BB/\text{mV})$ where BB is the barrier gate voltage modulating exchange at a balanced double-dot potential. As shown in Supplementary Fig. 1f, we extract $\alpha = 0.18$ for exchange tunability in the model. This can be compared to the measured relations $\alpha_1 = 0.04$, $\alpha_2 = 0.10$, $\alpha_3 = 0.06$, and $\alpha_4 = 0.09$ for bus stops 1 to 4 respectively. The reasonable qualitative agreement provides a basis for improving design rules that will become increasingly relevant for larger device designs.

Based on our observations of relatively modest two-qubit gate fidelities in comparison to the state of the art, we can comment on two possible design modifications for improved performance. In many instances, the barrier lever arms are larger than those observed in previous demonstrations of high-fidelity two-qubit gates. For instance, in [3], $\alpha = 0.02$ was observed, meaning that the exchange coupling in the present device is 2-5 times more sensitive to barrier voltage fluctuations. This, in tandem with the relatively modest attenuation on the barrier control lines, makes the spin qubits in the present device comparatively more sensitive to incoherent noise in the exchange strength. Altering the shape of the bus stop barrier gates to be smaller, or placing them in a higher metallization layer, would ameliorate this issue at the expense of requiring a more careful choice of DC voltage due to the less sensitive exchange tunability.

Supplementary Note 2. Magnetostatic design

The micromagnet is deposited with a window shape (see Fig. 1a) such that its stray field in the quantum well gives rise to a spin Hamiltonian that is practical for measuring and interacting qubits in the sparse array while being reasonably robust to small fabrication defects and microscopic variation. The design criteria are to permit single-spin control with EDSR at all sites in the array with addressability while maintaining resilience against electric charge noise. EDSR controllability enters the Hamiltonian through $b_\perp$, the magnetic field gradient transverse to the total field vector at each potential quantum dot location, and charge noise susceptibility is quantified by $b_\parallel$, the longitudinal magnetic field gradient parallel to the total field vector. $b_\parallel$ also gives rise to addressability between spins and the Zeemen energy differences required for high-fidelity parity-mode PSB readout [4] and fast adiabatic exchange interactions for two-qubit gates [5].

In order to calculate the relevant quantities, the point-like locations of the relevant quantum dots are extracted from electrostatic simulations as described in Sec. Supplementary Note 1, qualitatively approximating the dot positions in the experiment. We use the magpylib Python package to estimate the stray field of the micromagnet assuming homogeneous polarization, which is an approximation [6]. We use a magnet polarization of 730 mT to match the simulated qubit Larmor frequencies to the values measured in experiment. As the saturation polarization for cobalt micromagnets has been observed in the range of 1.5 T to 1.8 T, the magnet is partially demagnetized. However, we do not observe signs of micromagnet instability over several months of experiments. A detailed description of extracting the relevant quantities can be found in [7].

Supplementary Fig. 2a shows the total magnetic field in the quantum well, expressed as the spin Larmor frequency $f_\text{L}(x, y)$ assuming a g-factor of 2. The theoretical distribution shown in Fig. 1c is taken along the dashed line, while the estimates for quantum dots localized in the bus stops are taken using the maximum of the simulated single-particle spatial wave functions. For three of the four bus stops, we measure the qualitatively-expected Zeeman energy difference between bus and bus stop spins. Bus stop 4 is an exception, where the bus stop spin is lower in frequency than the bus, and the Larmor frequencies only differ by about 20 MHz. This requires a slower two-qubit interaction to maintain adiabaticity. The exact reason for this discrepancy presumably depends on the microscopics of the device which we cannot deduce with certainty. The micromagnet design is quite robust to misalignments with the external field (i.e. the behavior is nearly unchanged if the magnetization vector does not point exactly along the y-axis). Therefore, it may be a result of either magnetic domain alignment in the micromagnet, which our simulations do not account for; a misalignment in the deposition of the micromagnet; or a shift in the actual position of the spins with respect to their estimated locations on the order of tens of nanometers along the y-axis. In fact, we rely on some degree of disorder, as the nominal micromagnet design with homogeneous polarization gives rise to a perfectly symmetric profile such that data qubits 1 and 4 as well as 2 and 3 should have identical frequencies. It is anticipated from previous experiments that microscopic variation will lift this degeneracy on the order of tens of megahertz, which is what we observe in experiment.

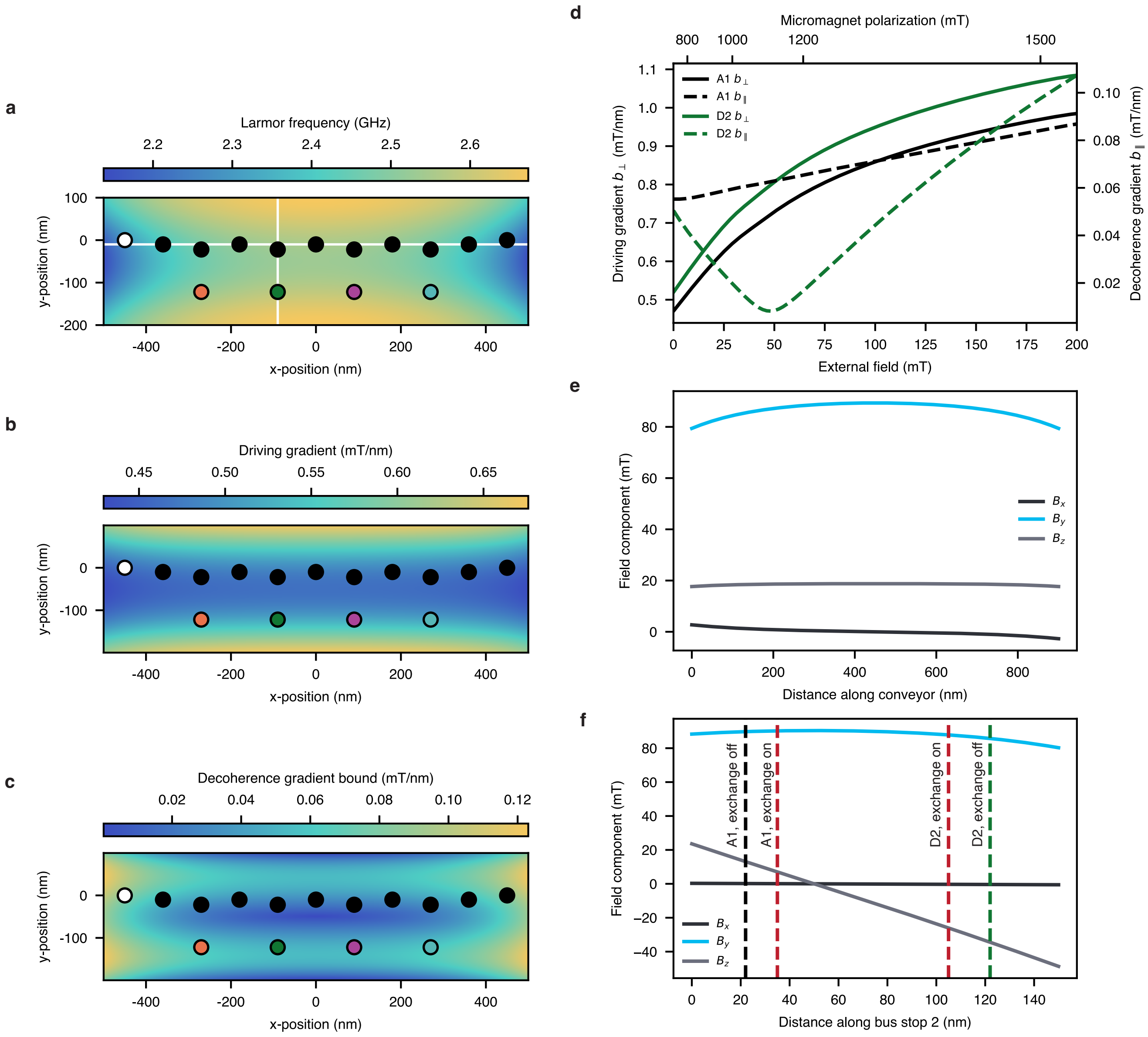

Supplementary Figure 2. **a** Simulated Larmor frequencies of an electron spin (with $g = 2$) in the quantum well when $B_{\text{ext}} = 0$ after partial polarization of the micromagnet. Points represent estimated locations of the spin localized along the shuttling bus and the bus stops as a guide to the eye. **b** Simulated driving gradient in the quantum well when $B_{\text{ext}} = 0$ after partial polarization of the micromagnet, assuming a control field oriented along the y-axis. **c** Simulated decoherence gradient in the quantum well when $B_{\text{ext}} = 0$ after partial polarization of the micromagnet. **d** The estimated impact of the external field setting, and therefore micromagnet polarization, on the driving and decoherence gradients at two representative quantum dot locations in the array. A1 is assumed to be localized below gate P2, as this is where it is resonantly controlled and idles in our experiments. Generally, the driving gradient decreases monotonically with external field, while the decoherence gradient may exhibit non-monotonic behavior. The homogeneous polarization of the micromagnet, in units mT, is phenomenologically fit to $1.155 \times 10^{-4}x^3 - 5.515 \times 10^{-2}x^2 + 10.38x + 734.1$ [mT] where $x = B_{\text{ext}}/\text{mT}$ based on the measured Larmor frequencies of R1 and A1 as the external field is lowered from 200 mT to zero. **e** Simulated magnetic field components along the shuttling bus axis as indicated by the horizontal white line in **a** when $B_{\text{ext}} = 0$ after partial polarization of the micromagnet. The maximum estimated tip in magnetic field vector along this axis is 2°. **f** Simulated magnetic field components along the double-dot potential of bus stop 2 as indicated by the vertical white line in **a**. The vertical dashed lines indicate estimated spin qubit locations depending on whether the exchange interaction is pulsed on or off. The estimated tip in magnetic field vector between conveyor and bus stop dots ranges between 30° when exchange is off and 20° when exchange is on.

The estimated transverse gradient $b_\perp(x, y)$ for driving along the y-axis is shown in Supplementary Fig. 2b and is dominated by the $\partial B_z(x, y)/\partial y$ gradient component. It is relatively constant over the sparse array, and we expect similar Rabi frequencies $f_{\rm R}$ to be achieved for equivalent driving amplitudes for all qubits. As $f_{\rm R}$ also depends on the microscopic dipole moment of the driven quantum dots (Methods), the driving amplitudes required to achieve a constant $f_{\rm R}$ across the array typically vary by an order-unity factor.

The longitudinal gradient $b_\parallel(x, y)$ through which electric noise couples to the spin depends on the spatial orientation of the fluctuation. We therefore take $|\nabla f_{\rm L}(x, y)|$ as an estimate of the noise susceptibility, which will consist of primarily $\partial B_y(x, y)/\partial x$ and $\partial B_y(x, y)/\partial y$ components. Supplementary Fig. 2c shows the sweet spot that emerges along the x-axis of symmetry. While this sweet spot cannot be shared by both the bus axis and the bus stops, in all cases the gradient remains below 0.1 mT/nm which is considered reasonable [8, 9].

Since the superconducting solenoid we used cannot be operated in persistent mode, the device is operated with only the remanence field of the micromagnet to avoid magnetic noise arising from current noise in the supply that drives the solenoid. The inclusion of a noiseless external field would have meaningful consequences. Two illustrative cases are plotted in Supplementary Fig. 2d for the ancilla qubit A1 localized below P2 and data qubit D2 localized below BP2. In general, a monotonic decrease in driving gradient is observed as the external field is lowered to zero. The decoherence gradient generally decreases as the external field is lowered from 200 mT, but the trend is not necessarily monotonic and the minimum may not be located at zero external field. In fact, a performance boost would likely follow from operating with a noiseless external field of 50 mT oriented along the y-axis (instead of zero external field) as both the Rabi frequency should increase and most qubits would be more robust to charge noise.

Supplementary Fig. 2e shows the magnetic field components along the shuttling bus and highlights the homogeneity of the estimated Larmor vector across the array. The orientation of the magnetic field vector at one end of the conveyor differs from that at the center by only about 2° which facilitates fast adiabatic spin shuttling. Supplementary Fig. 2f shows the magnetic field components along the axis of bus stop 2 that are relevant for the two-qubit interaction taking place there. A sizable tip of about 25° exists between the magnetic field vectors at each quantum dot location. Such a tip gives rise to both spin-conserving and spin-flipping tunnel coupling between quantum dots. Along with the moderate expected Zeeman energy difference of about 50 MHz between the interacting qubits, this makes the adiabatic controlled-phase gate more appealing to implement than other possible two-qubit interactions. If the external field were made to oppose the micromagnet polarization, the spin Larmor frequencies would decrease and the tip angle between bus stop and shuttling bus dots would increase, and baseband hopping control at the bus stop locations would also be possible [7, 10].

Supplementary Note 3. Towards logical Loss-DiVincenzo spin qubits

Here, we summarize the additional steps required to realize a complete $[\![4, 2, 2]\!]$ error-detecting code to illustrate how the weight-four parity checks demonstrated in this work can be used to construct logical qubits based on Loss-DiVincenzo spin qubits. We highlight that this code was recently demonstrated in silicon using exchange-only spin qubits [11] as well as donor-based spin qubits [12]. Supplementary Fig. 3a illustrates the conceptual connectivity of the rotated toric code with two plaquettes and its associated logical operators [13]. Supplementary Fig. 3b illustrates the corresponding spin qubit locations. The readout zone on the right side of the image functions analogously to the one on the left, where a stationary readout ancilla allows for the mobile ancilla qubit to be measured in the computational basis. The two sensors are connected to tank circuits with different resonance frequencies, allowing each SET conductance to be measured in parallel.

Mid-circuit measurements are possible in the present device, though we have not characterized them fully. They require dynamical decoupling of the data qubits while PSB and charge sensing take place, and there is a trade-off between dephasing and the measurement SNR. In our experiments, we use a measurement time of 10 µs as this permits a high SNR, and only daily calibrations are necessary to maintain a single-shot threshold. However, high-quality readout is possible in this device with measurement times as short as 5 µs. We anticipate that an optimal measurement time would exist within this range, and choose 8 µs as a reasonable estimate. High-fidelity PSB readout in a similar device has been achieved in below 3 µs [14].

The two mobile ancillas may be used to extract the two weight-four stabilizers necessary to infer a complete error syndrome in the $[\![4, 2, 2]\!]$ encoding. Supplementary Fig. 3c illustrates the compiled quantum circuit that can be used to extract both bits of information. In our experiment, the weight-four Z-type check took place in 4.84 µs and the weight-four X-type check took place in 5.84 µs owing to the additional single-qubit gates required to change basis. A compiled extraction of both stabilizers would therefore require 10.43 µs using the experimental shuttling speed of 1.8 m/s. By increasing the shuttling speed to 50 m/s, which has previously been demonstrated with high fidelity [15], each syndrome extraction could be up to 1.4 µs faster, with the total circuit duration dropping to below 8 µs. At this point, the single-qubit gates form the largest time bottleneck, and the circuit duration could be further reduced by

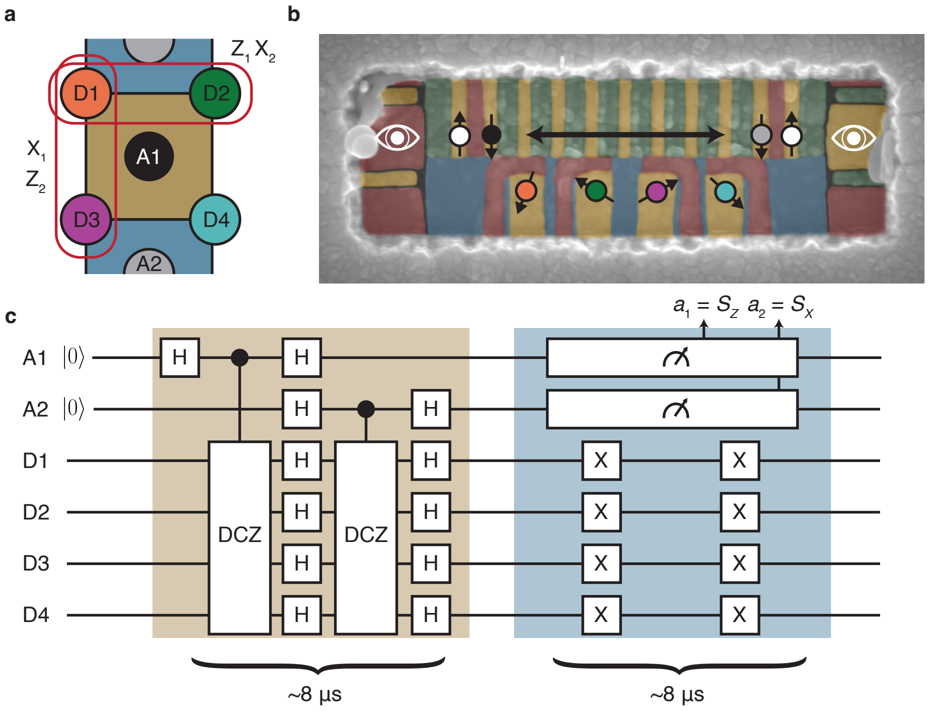

Supplementary Figure 3. **a** Illustration of a ⟦4, 2, 2⟧ rotated toric code. Ancilla qubits A1 and A2 correspond to stabilizer plaquettes measuring $S_Z = ZZZZ$ and $S_X = XXXX$ respectively. Logical operations on the 2 encoded qubits are indicated. **b** False-colored scanning electron microscope image depicting the data qubit and ancilla locations when the device is operated as a quantum memory. **c** Quantum circuit depicting a complete syndrome extraction and measurement. The DCZ operation is realized as depicted in Fig. 4a and includes dynamical decoupling. The duration of the circuit is estimated based on the times reported in Extended Data Fig. 10a, where it is further assumed that the shuttling time can be reduced from 730 ns to 100 ns or faster. Single-qubit gates are assumed to be parallelized on pairs of qubits as was done in this work.

decreasing the single-qubit gate time or simultaneously driving more qubits at once. We remark that high-fidelity driving of five qubits using the same EDSR mechanism as in this work has already been demonstrated [16].

We therefore estimate, conservatively, that a complete round of error detection for a ⟦4, 2, 2⟧ code may take place in about 16 µs, during which four sets of refocusing pulses are applied to all data qubits. Following the coherence measurements of Fig. 3e, dynamical decoupling at this rate corresponds to an average data qubit coherence of 334 µs, and we can expect to perform about 20 rounds of error detection within this time. Such a demonstration would compare with initial implementations of error detection with superconducting qubits [17].

The primary obstacle to realizing effective quantum error detection in the present device are the relatively low two-qubit gate fidelities, but, as summarized in the main text, we believe that minor modifications in device and setup design can raise these to state-of-the-art values, which exceed 99% using the same device physics and control methods employed here [3, 18]. High-fidelity shuttling [15], simultaneous single-qubit gates [16], and readout [14] have similarly been demonstrated, including at speeds faster than estimated here. The consolidation of all of these performance benchmarks in the present shuttling architecture would enable achievable implementations of logical Loss-DiVincenzo spin qubits.

To make a first-order estimate of a plausible total success probability for a complete syndrome extraction, we take the following state-of-the-art metrics: shuttling 1.2 µm in 25 ns with an error rate of 0.001 [15]; two-qubit gates with an error rate of 0.004 with the same duration as in our work [3]; single-qubit gates with an error rate of 0.0002 and the same duration and parallelization as in our work; and measurement in 3 µs with an error rate of 0.005 [14]. We assume both ancillas have the same coherence properties and use the CPMG-4 coherence measurements to account for the effect of dephasing during the circuit of Supplementary Fig. 3c. Using the same approach as for the error budget described in the Methods, we estimate a 84% success probability of correct syndrome extraction and data qubit state preservation. We note that the majority of this error arises due to data qubit idling, and this is the case even for two-qubit error rates as high as about 1.5%. Further improvement will require a combination of shortening the measurement time, minimizing the gate durations, and improving the physical coherence properties of the spins.

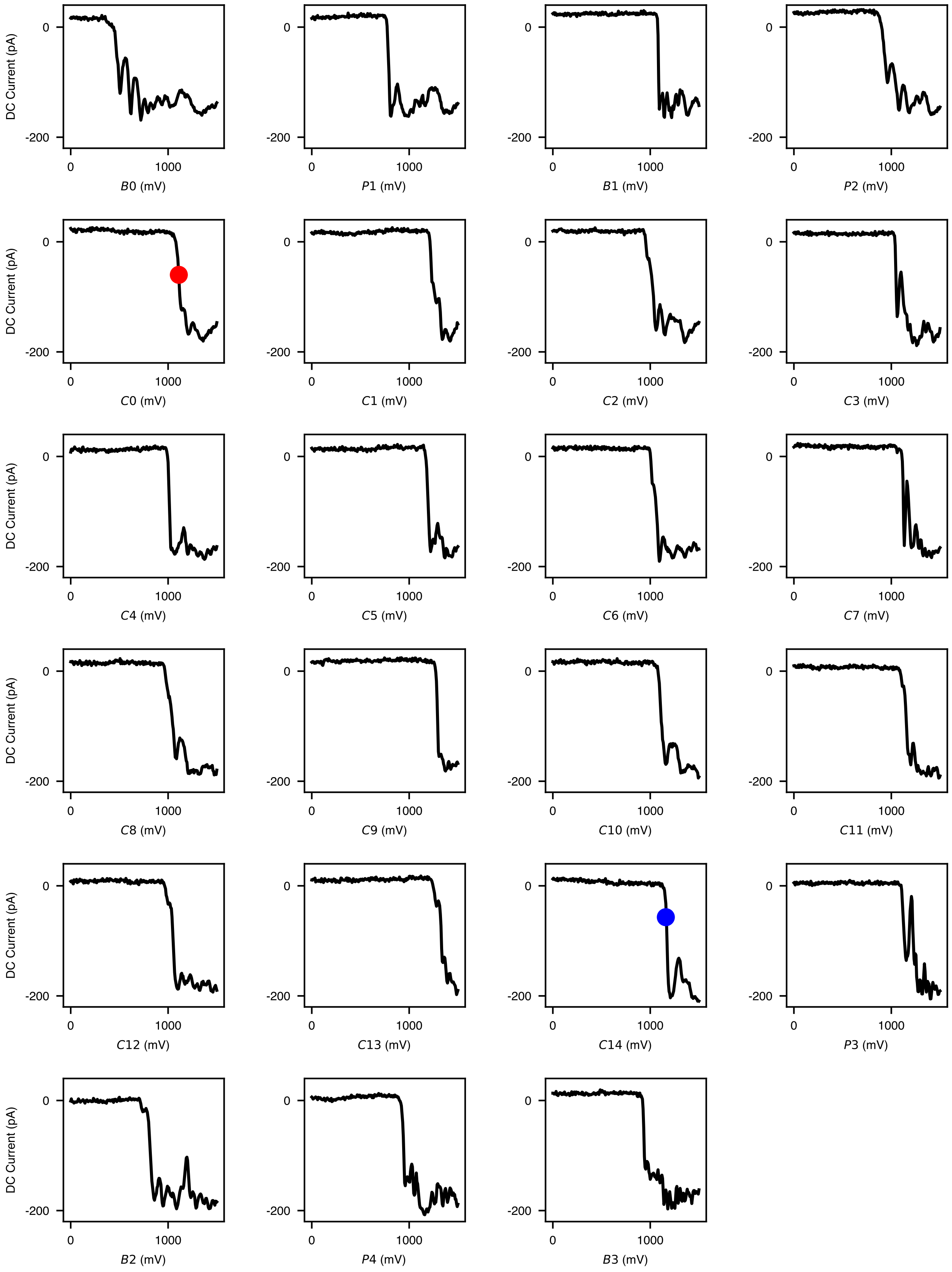

Supplementary Figure 4. Collection of turn-on voltages measured during initial sample screening. We use a negative bias voltage of $-100\,\mu V$ from source to drain to differentiate the measured current from an undesired positive leakage current that passes through the swept gate. The colored points indicate the settings used to test bus stop gates. The data presented is from the sample which was subsequently used for all experiments in this work.

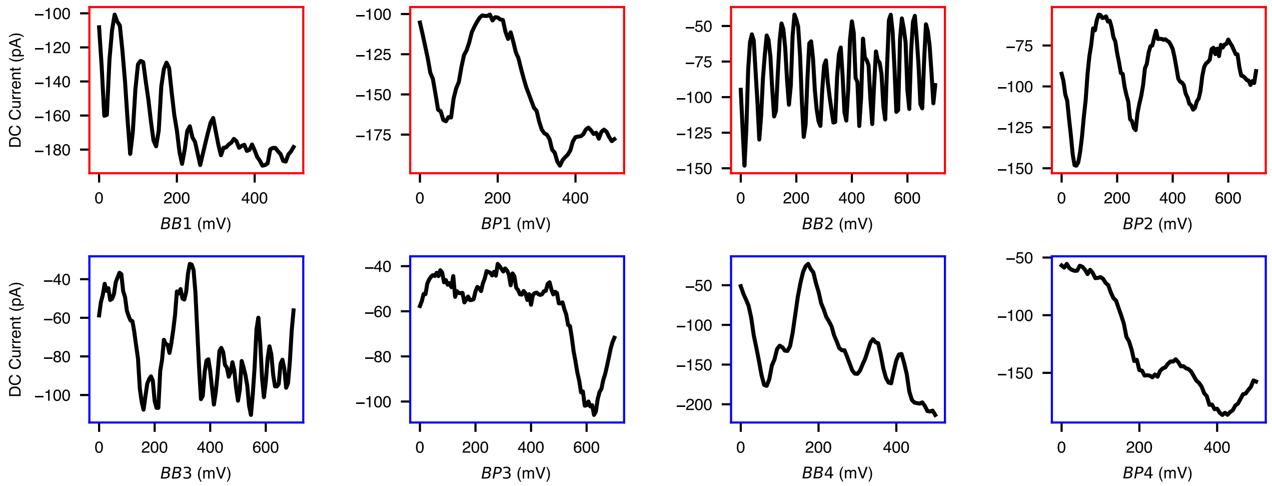

Supplementary Figure 5. Collection of DC voltage sweeps for all 8 bus stop gates to test their effect on conduction through the conveyor channel. For bus stops 1 and 2, $C0$ is set to 1.15 V as shown in Supplementary Fig. 4 while all other gates are accumulated in order to create an electrostatically sensitive channel near the tested gates. For bus stops 3 and 4, $C14$ is set to 1.18 V by the same reasoning.

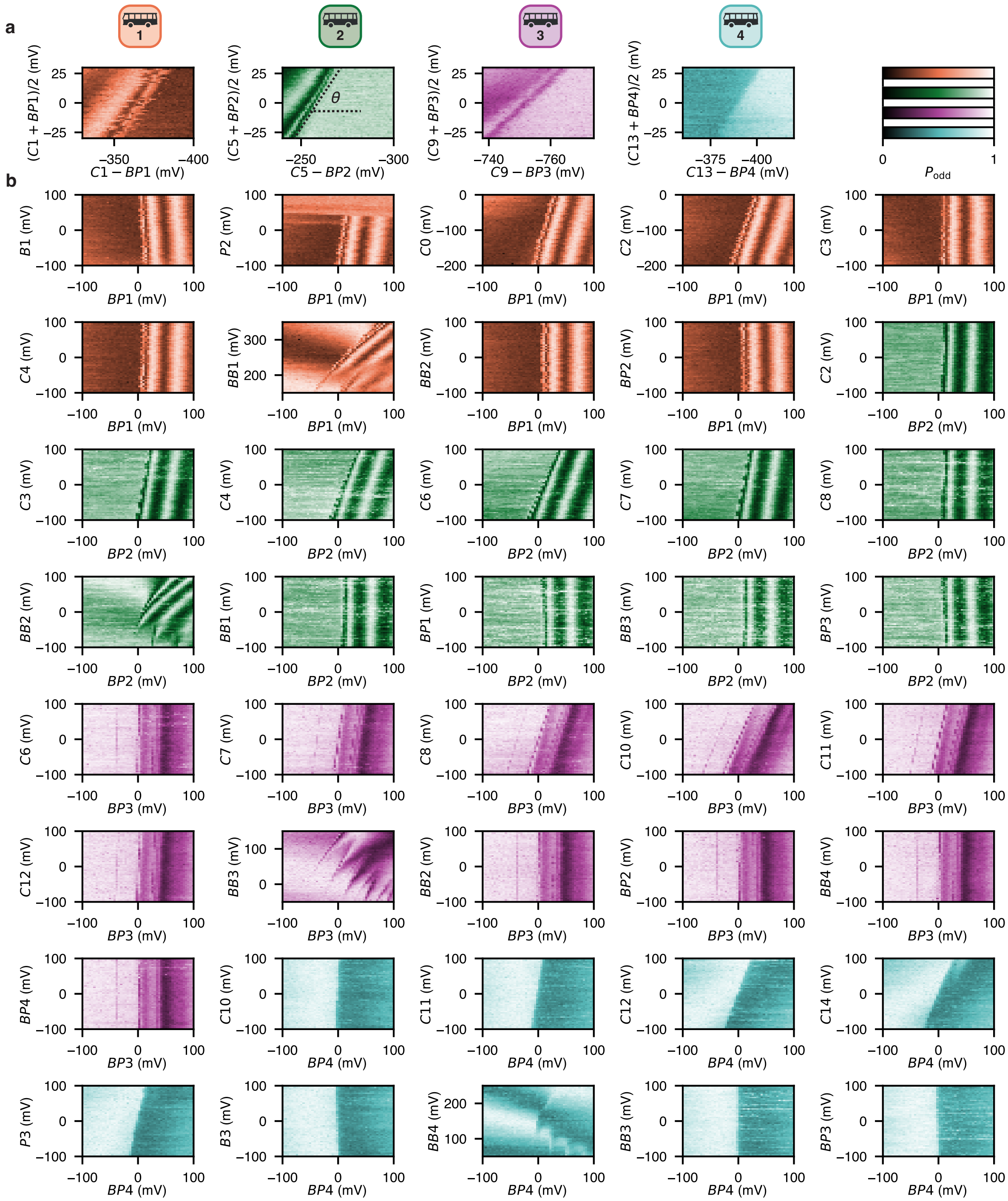

Supplementary Figure 6. **a** Single-electron charge stability diagrams measured via the phase pick-up of a tunneled spin in superposition for each of the four bus stops. The four bus stops are color-coded for clarity, but the measured signal is the probability of an odd-parity PSB outcome P_{odd} in all cases. The angle θ, which allows for calibration of the virtual detuning and average potential as described in the Methods, is labeled. **b** Analogous gate-gate virtualization measurements for all gates G in the vicinity of each bus stop. The extracted slopes $\mathrm{d}BPi/\mathrm{d}G$ from all scans are represented as a heat map in Fig. 2g.

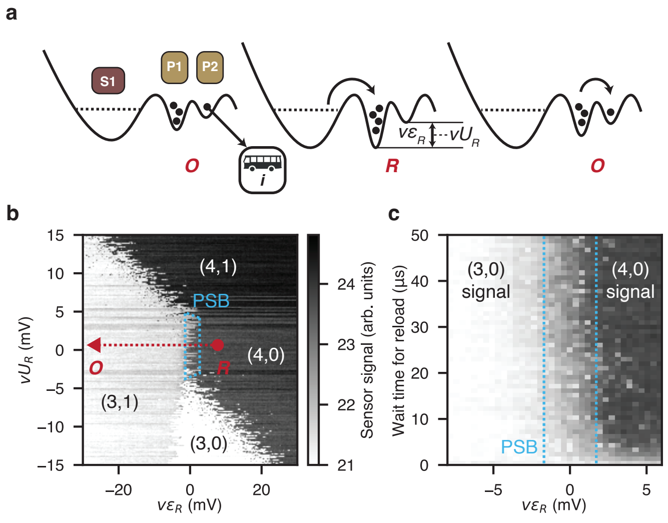

Supplementary Figure 7. **a** Illustration of the reload protocol. After shuttling the ancilla spin to a bus stop, the charge state of the readout pair is blockaded in the metastable (3,0) charge state at the operation point O. When the detuning is pulsed to the reload point R, the blockade is lifted and a fourth electron quickly tunnels from S1 to the readout ancilla dot. The double-dot system returns to the (3,1) charge state upon ramping the detuning back to the point O. **b** rf-reflectometry measurement of the equilibrium charge stability diagram of the readout pair. The PSB region is highlighted by the blue dashed lines. The detuning axis followed to go from the operation point O to the reload point R is shown by the red dashed line. **c** Averaged rf-reflectometry time-traces after waiting at different reload points along the detuning axis of **b** after the system has been initialized in the metastable (3,0) charge state. Between O and the PSB region, blockade persists for at least 20 ms. Beyond the PSB region, the reload time scale is on the order of 10 µs. The colorbar is identical to that of **b**.

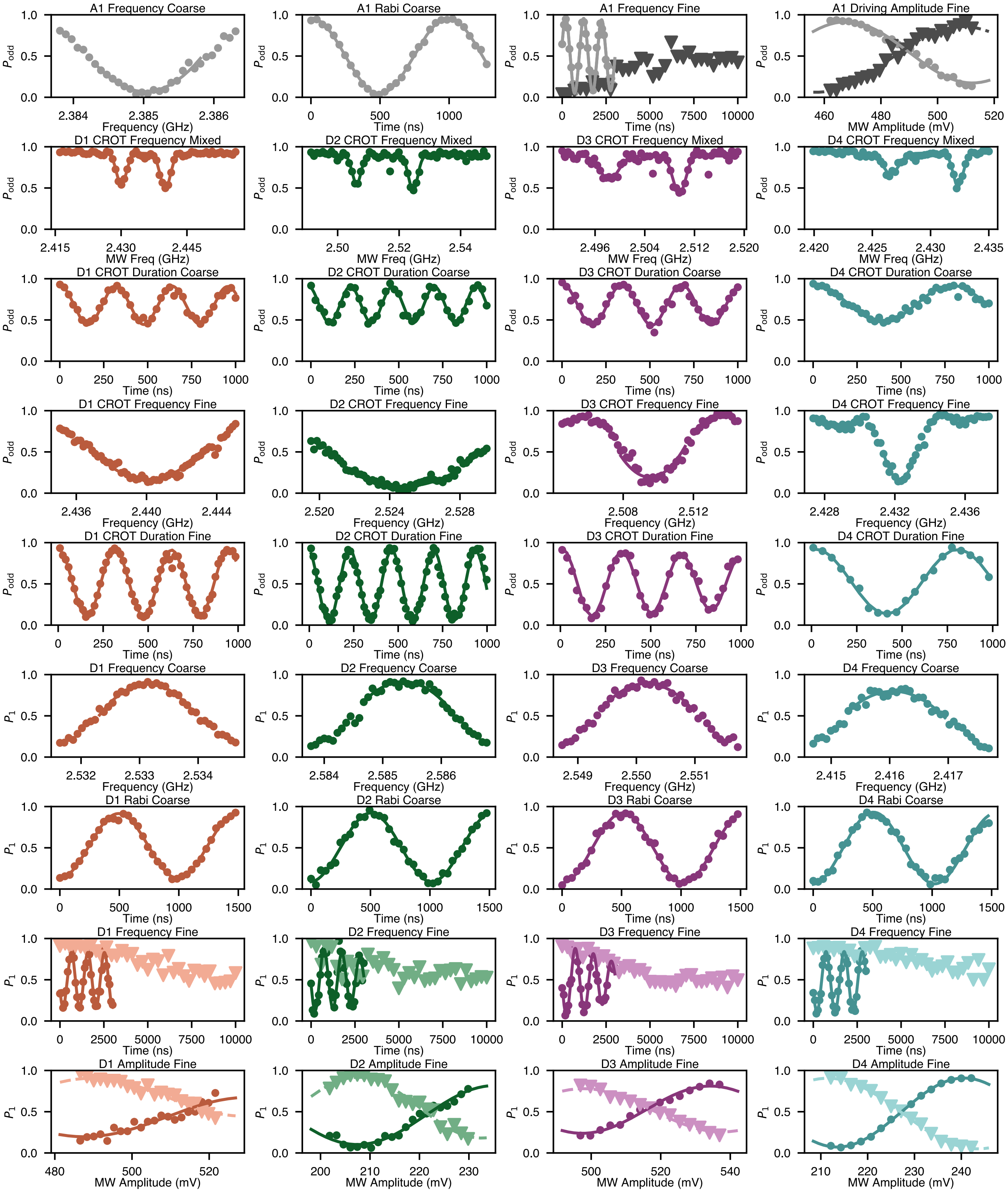

Supplementary Figure 8. A calibration report for all resonant operations. The order of the experiments is from left-to-right and top-to-bottom. For fine frequency experiments, circles indicate a scan with a virtual detuning, and triangles indicate a verification scan. For fine driving amplitude experiments, circles and triangles indicate whether a final X_{-90} or X_{90} gate was applied. Ancilla measurements are reported as the probability P_{odd} of an odd-parity PSB outcome. Data qubit QND measurements are reported as the probability P_1 of inferring the $|1\rangle$ state.

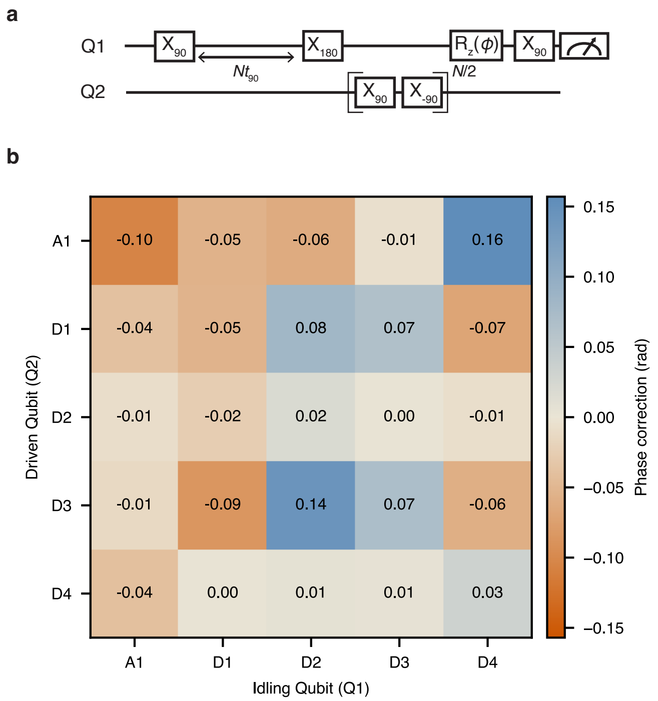

Supplementary Figure 9. **a** Quantum circuit used to perform a Hahn-echo-type experiment and characterize the single-qubit phase crosstalk accrued by Q1 while performing single-qubit operations on Q2. **b** An example of the crosstalk phase corrections that are applied along with each corresponding X_{90} gate while operating the device as a five-qubit processor.

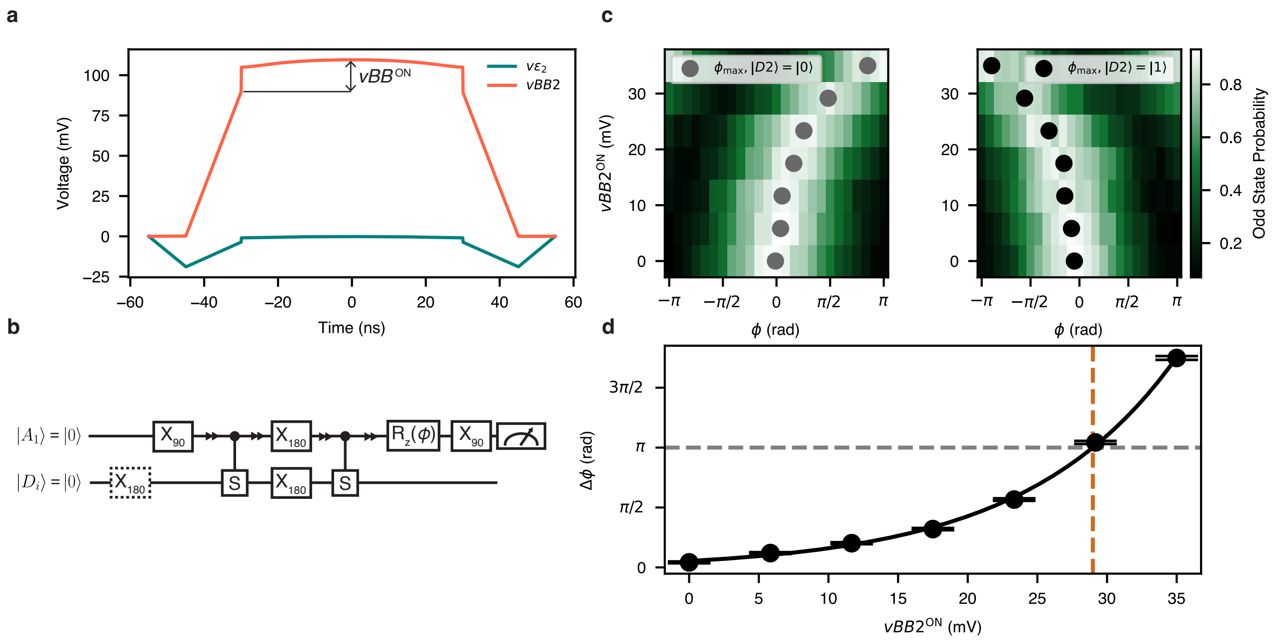

Supplementary Figure 10. **a** An example of a Hamming-window exchange pulse for implementing a CS gate between A1 and D2. **b** Quantum circuit used to fine-tune the maximum amplitude of the Hamming pulse. **c** The phase pickup of the ancilla qubit for different maximum Hamming pulse amplitudes for both computational-basis preparations of the data qubit D2. The total gate time is fixed. The markers indicate the relative shift of the curves due to conditional phase pickup. **d** The difference in the conditional phase picked up by the ancilla is fit and we select the barrier amplitude that yields a total phase difference of π radians from the two successive CS gates.

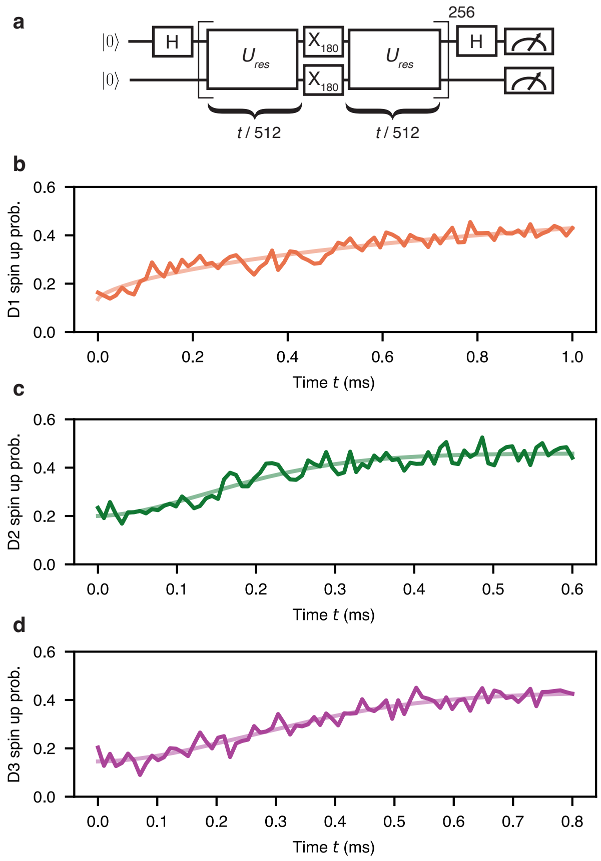

Supplementary Figure 11. **a** Circuit to measure the residual exchange between two data qubits localized in bus stops. The unitary evolution under idling is Ising-like as $J_{\mathrm{res}} < \Delta E_{\mathrm{Z}}$, and $U_{\mathrm{res}} \approx \mathrm{diag}\left(1, e^{iJ_{\mathrm{res}}t/2}, e^{iJ_{\mathrm{res}}t/2}, 1\right)$, therefore the oscillations of the qubit placed in superposition will have a frequency $J_{\mathrm{res}}/2$ as a function of the total idling duration. **b-d** Measurements of residual exchange between D1 and D2, D2 and D3, and D3 and D4 respectively. In all cases, no residual exchange on the order of kilohertz is discernible from decoherence and is therefore negligible for any experiment conducted in this work. Due to the relatively large separation and large potential barrier between data qubits, the actual residual exchange is likely much lower.

Bus stop	Qubits	F_{ref} (%)	F_{DCZ} (%)	F_{Dsh} (%)
1	A1 and D1	96.2(2)	84.2(8)	91.9(5)
2	A1 and D2	97.4(3)	87.9(6)	93.0(4)
3	A1 and D3	96.7(2)	82.8(1.3)	92.6(3)
4	A1 and D4	97.7(1)	89.3(5)	93.9(8)

Supplementary Table I. Table of extracted fidelities using interleaved CRB. The reference fidelity F_{ref} corresponds to the fidelity of simultaneous single-qubit Clifford gates in the relevant two-qubit Hilbert space. The DCZ operation with fidelity F_{DCZ} is equivalent to the two-qubit version of the circuit displayed in Fig. 4a. The decoupled shuttling operation with fidelity F_{Dsh} is the same two-qubit operation but with the entangling CS interactions replaced by idling instead.

[1] F. Beaudoin, P. Philippopoulos, C. Zhou, I. Kriekouki, M. Pioro-Ladrière, H. Guo, and P. Galy, Robust technology computer-aided design of gated quantum dots at cryogenic temperature, Applied Physics Letters **120**, 264001 (2022).

[2] G. Burkard, T. D. Ladd, A. Pan, J. M. Nichol, and J. R. Petta, Semiconductor spin qubits, Rev. Mod. Phys. **95**, 025003 (2023).

[3] X. Xue, M. Russ, N. Samkharadze, B. Undseth, A. Sammak, G. Scappucci, and L. M. K. Vandersypen, Quantum logic with spin qubits crossing the surface code threshold, Nature **601**, 343–347 (2022).

[4] A. E. Seedhouse, T. Tanttu, R. C. Leon, R. Zhao, K. Y. Tan, B. Hensen, F. E. Hudson, K. M. Itoh, J. Yoneda, C. H. Yang, A. Morello, A. Laucht, S. N. Coppersmith, A. Saraiva, and A. S. Dzurak, Pauli blockade in silicon quantum dots with spin-orbit control, PRX Quantum **2** (2021).

[5] M. Rimbach-Russ, S. G. J. Philips, X. Xue, and L. M. K. Vandersypen, Simple framework for systematic high-fidelity gate operations, Quantum Science and Technology **8**, 045025 (2023).

[6] M. Aldeghi, R. Allenspach, A. Vervelaki, D. Jetter, K. Bagani, F. Braakman, M. Poggio, and G. Salis, Simulation and measurement of stray fields for the manipulation of spin qubits in one- and two-dimensional arrays, Nano Letters **25**, 1838 (2025).

[7] F. K. Unseld, B. Undseth, E. Raymenants, Y. Matsumoto, S. L. de Snoo, S. Karwal, O. Pietx-Casas, A. S. Ivlev, M. Meyer, A. Sammak, M. Veldhorst, G. Scappucci, and L. M. K. Vandersypen, Baseband control of single-electron silicon spin qubits in two dimensions, Nature Communications **16**, 5605 (2025).

[8] J. Yoneda, K. Takeda, T. Otsuka, T. Nakajima, M. R. Delbecq, G. Allison, T. Honda, T. Kodera, S. Oda, Y. Hoshi, N. Usami, K. M. Itoh, and S. Tarucha, A quantum-dot spin qubit with coherence limited by charge noise and fidelity higher than 99.9%, Nature Nanotechnology **13**, 102–106 (2017).

[9] S. G. J. Philips, M. T. Mądzik, S. V. Amitonov, S. L. de Snoo, M. Russ, N. Kalhor, C. Volk, W. I. L. Lawrie, D. Brousse, L. Tryputen, B. P. Wuetz, A. Sammak, M. Veldhorst, G. Scappucci, and L. M. K. Vandersypen, Universal control of a six-qubit quantum processor in silicon, Nature **609**, 919–924 (2022).

[10] C.-A. Wang, V. John, H. Tidjani, C. X. Yu, A. S. Ivlev, C. Déprez, F. van Riggelen-Doelman, B. D. Woods, N. W. Hendrickx, W. I. L. Lawrie, L. E. A. Stehouwer, S. D. Oosterhout, A. Sammak, M. Friesen, G. Scappucci, S. L. de Snoo, M. Rimbach-Russ, F. Borsoi, and M. Veldhorst, Operating semiconductor quantum processors with hopping spins, Science **385**, 447–452 (2024).

[11] Members of the HRL Quantum Team and Collaborators, A digitally controlled silicon quantum processing unit (2026), arXiv:2604.16216 [quant-ph].

[12] C. Zhang, C. Li, Z. Tian, Y. Jiang, F. Xu, S. Zhang, H. Wang, Y.-N. Zhang, X. Bai, B. Zhao, Y.-F. Zhang, H. Shu, J. Liu, K. Wu, C. Huang, K. Shi, M. Duan, T. Xin, P. Huang, T. Pan, S. Liu, G. Wang, G. Hu, Y. He, and D. Yu, Quantum error detection in a silicon quantum processor, Nature Electronics , 1–9 (2026).

[13] N. M. Linke, M. Gutierrez, K. A. Landsman, C. Figgatt, S. Debnath, K. R. Brown, and C. Monroe, Fault-tolerant quantum error detection, Science Advances **3** (2017).

[14] K. Takeda, A. Noiri, T. Nakajima, L. C. Camenzind, T. Kobayashi, A. Sammak, G. Scappucci, and S. Tarucha, Rapid single-shot parity spin readout in a silicon double quantum dot with fidelity exceeding 99%, npj Quantum Information **10** (2024).

[15] M. De Smet, Y. Matsumoto, A.-M. J. Zwerver, L. Tryputen, S. L. de Snoo, S. V. Amitonov, S. R. Katiraee-Far, A. Sammak, N. Samkharadze, O. Gül, R. N. M. Wasserman, E. Greplová, M. Rimbach-Russ, G. Scappucci, and L. M. K. Vandersypen, High-fidelity single-spin shuttling in silicon, Nature Nanotechnology **20**, 866–872 (2025).

[16] Y.-H. Wu, L. C. Camenzind, P. Bütler, I. K. Jin, A. Noiri, K. Takeda, T. Nakajima, T. Kobayashi, G. Scappucci, H.-S. Goan, and S. Tarucha, Simultaneous high-fidelity single-qubit gates in a spin qubit array (2025), arXiv:2507.11918 [quant-ph].

[17] C. K. Andersen, A. Remm, S. Lazar, S. Krinner, N. Lacroix, G. J. Norris, M. Gabureac, C. Eichler, and A. Wallraff, Repeated quantum error detection in a surface code, Nature Physics **16**, 875–880 (2020).

[18] A. R. Mills, C. R. Guinn, M. J. Gullans, A. J. Sigillito, M. M. Feldman, E. Nielsen, and J. R. Petta, Two-qubit silicon quantum processor with operation fidelity exceeding 99%, Science Advances **8** (2022).
