# Supplementary material for: Weight-four parity checks in a spin-shuttling architecture
Source: Nature. 2026 Jul 29;655(8125):1160–6. doi: 10.1038/s41586-026-10766-3 (PMC13421349; doi:10.1038/s41586-026-10766-3)
Supplement: Supplementary file 2 — Peer Review File [file 41586_2026_10766_MOESM2_ESM.pdf]

nature portfolio

Peer Review File

Weight-four parity checks in a spin-shuttling architecture

Corresponding Author: Mr Brennan Undseth

Any redactions in this file are there to maintain patient confidentiality, the confidentiality of unpublished data, or to remove third-party material.

This file contains all reviewer reports in order by version, followed by all author rebuttals in order by version.

Version 0:

Reviewer comments:

Referee #1

(Remarks to the Author)
This tour-de-force paper describes the operation of a sparse spin-qubit array in silicon. Using spin shuttling, the authors demonstrate weight-four parity checks and the generation of a 5-qubit GHZ state. The device design, tune-up, and operation are novel, and the demonstration of parity checks in a semiconductor spin-qubit platform is a major step forward. The experiments are very well done and clearly described, and the paper likely suitable for Nature. Here are my questions:
1. Looking ahead, what are the main improvements, if any, that would need to be made to demonstrate elementary QEC with this platform? In addition to the state preparation fidelity, I also wonder about the time it takes to implement the parity check, how this compares with the expected coherence time of the physical qubits, and to what extent this matters for fault-tolerant QEC.
2. Are the relatively low 2Q fidelities consistent with the observed levels of electrical noise, or is there another cause? What is the most likely method for improvement? Can the authors speculate on why relatively low 2Q fidelities were observed here in contrast to the higher 2Q gate fidelities in earlier work?
3. Do the extracted check errors also agree with expectations? The relatively simple error analysis for the GHZ state generation agrees with the calculated fidelities, but does this also hold for the check itself?
4. The distribution of values for both the check accuracy and entangled state fidelity seem to significantly exceed the reported uncertainties. Why is this the case? Is this related to the experiment or the analysis?
5. Can the authors briefly describe the full tune-up process, from the beginning?

Referee #2

(Remarks to the Author)
Undseth et al. present an impressive set of results showcasing the utility of coherent spin shuttling in increasingly complex semiconductor quantum devices. The manuscript goes well beyond prior work that only characterized the coherence of the shuttling protocol or used it as a local probe of valley splitting. The authors first describe a new device that incorporates readout, spin conveyor, and qubit zones. Figure 2 nicely showcases the capabilities of spin shuttling - using just a single charge detector at the left end of the device, the authors are able to remotely tune-up qubits located as "bus stops" 1 - 4. The full loading, initialization, control and readout sequence is detailed in Fig. 3. Finally, the authors demonstrate weight-4 parity checks and the synthesis of a 5 qubit GHZ state in Fig. 4. The research is essentially moving the spin qubit community towards a significant quantum error correction demonstration, e.g. the [[4,1,2]] code.

Overall, the manuscript is of the highest quality and is most suitable for publication in Nature. On the whole, this research and other upcoming research by HRL as well as Diraq/imec are poised to bring spin qubits neck-and-neck with superconducting qubits. I therefore believe this is an important result that will reshape the perception of spin qubits in the broader community of quantum computing researchers.

My only critical comment relates to performance metrics and projections for future work. While the work is impressive, the parity check accuracy of 67-73% is far below the commonly cited 99% threshold for the surface code. Two qubit gate fidelities are vastly limiting the overall performance of the device (up to 10% error, much worse than the best demos >99%). I therefore encourage the authors to provide a more balanced outlook for future work - many performance improvements are

required before the basic ingredients contained in this demonstration can be put together to demonstrate a logical qubit. The final two paragraphs could be better put to use explaining how the remaining technological hurdles may be overcome.

Referee #3

(Remarks to the Author)
In “Weight-four parity checks with silicon spin qubits” the authors introduce a new Si/SiGe spin qubit device and architecture and lay ground work for future quantum error correction (QEC) demonstrations by demonstrating spin shuttling, quantum non-demolition spin measurements, parity-checks, and GHZ states of up to five-qubits.

This paper is a tour de force of gate-defined spin qubit design, tune-up, and operation that pushes forward the state of the art of that field in its first appearance (I think it’s the first) and deserves a high-visibility publication somewhere. In my opinion, the significance of this work is less the weight-four parity checks highlighted in the title than the new device architecture they demonstrate. Unfortunately, the gate fidelities are not high, falling quite short of what’s necessary for even break-even error correction — and so I would dial back on the word “immediate” in the statement “This work opens immediate opportunities to pursue QEC experiments with spin qubits.” The quantum information concepts here are well-explored and better performant in other quantum technologies and it’s less obvious to me what this work offers to those outside of the gate-defined spin qubit community. I would defer to other referees more further afield as to whether this work is of sufficient broad interest for Nature as opposed to one of its more specialized journals.

This device is the most ambitious shuttling-based spin qubit device that I’m aware of, which is the source of much of its power. As the authors highlight, shuttling adds flexibility to a spin qubit device design, facilitating effective long-range and reconfigurable interactions between data qubit sites. It also permits a sparser layout, which can be strategically employed to mitigate routing density and cross-talk challenges with this technology. Qubit shuttling is not unique to gate-defined spin qubits, and is a core characteristic of other quantum platforms, but perhaps this is the most capable demonstration in a solid-state platform. Specifically in this demonstration, the authors use the shuttling to realize a demonstration of a surface-code stabilizer plaquette and to do this on an extended device (9-dots-deep, in a sense) with only a single readout site.

The tune-up of this device with such a remote, single readout site is remarkable, with a number of innovations detailed in the main article and supplement. These descriptions take up much, maybe most, of the article, but are probably of less interest to those outside the field, I fear.

My biggest concern about the viability of the design — even in the immediate term — are the relatively low 2-qubit errors achieved. The authors point out that several similar devices have achieved 2-qubit errors significantly below 1% error, but all such errors in this device are between 10% and 5%, without explanation, suggesting some systemic issue in the design, processing, or control. What are some of the possibly relevant differences between this work and those other, higher performant ones? What evidence is there for a straightforward path for ab improvement of 10x or more without a significant change to the design or approach? For example — to simply pick a hypothetical trade to illustrate my general concern — if a lot of the issue is a too-small lever arm of the BB(X) gates because it’s higher in the stack, solving this issue by reordering the stack or changing the gate shape would likely trade with shuttling fidelity, or maybe some other figure of merit.

Similarly, while the shuttling fidelity is good — and the authors claim it’s the highest performant by some metrics — incurring a >2% error moving the ancilla around also needs to be improved at least 10x for error correction viability. The authors suggest vaguely that shuttling faster would improve the fidelity, but going faster always exacerbates other issues like control fidelity or state transitions. My general point is that although the authors demonstrate the functional ability of some key QEC operations, there’s not much evidence that the performance issues have a clear path to improvement without some unknown larger changes, and so even the short term viability of the design has questions.

Associated smaller questions and feedback:
Fig 1a: A silly aesthetic nit-pick, but the four “bus stops” are depicted as “buses,” while the shuttling electron is really the moving vehicle. Sorry, this might just be me. I might be the only person who would think about this.
Fig 1c: The legend doesn’t clearly specify the circles as either “simulation” or “measurement.” It can be deduced by some context that they are measurements, but there’s probably a clearer way to organize the legend.
Page 3: there is a comment that the slowest operation is PSB readout, which takes 10 us. Given that this long integration likely comes at other fidelity costs or complications, it might be illuminating to explain more in the supplement how this integration time was chosen.
Fig 3g: Why is the ancilla 1Q gate 10x worse than the data qubit 1Q gates?
Page 5: There is a comment that the ancilla is shuttled back from the D4 qubit to P2 to perform a higher fidelity 1Q decoupling pulse, but this would seem unlikely given the 2.3% fidelity hit this extra shuttling would incur? Do the ancilla 1Q gates not under P2 have 3% or worse error? Or am I misunderstanding something?
Page 6: The authors claim that this is the first observation of “genuine five-qubit entanglement” in a gate defined semiconductor spin, and while this may be true, the observed fidelity appears to be ~53%, which is quite marginal, especially considering the probability corrections in state tomography (no matter how common and an accepted, necessary evil such corrections may be). This claim cannot be given much weight, unfortunately.
Page 7: The authors claim this approach could tune up 100 qubits with a single charge sensor? What sets this limit? (e.g. Why not 1000?). What shuttling fidelity is this assuming, and why is this plausible?
Appendix G: What are the T1(0,1) times?

Open Access This Peer Review File is licensed under a Creative Commons Attribution 4.0 International License, which permits use, sharing, adaptation, distribution and reproduction in any medium or format, as long as you give appropriate credit to the original author(s) and the source, provide a link to the Creative Commons license, and indicate if changes were made.
In cases where reviewers are anonymous, credit should be given to 'Anonymous Referee' and the source.
The images or other third party material in this Peer Review File are included in the article's Creative Commons license, unless indicated otherwise in a credit line to the material. If material is not included in the article's Creative Commons license and your intended use is not permitted by statutory regulation or exceeds the permitted use, you will need to obtain permission directly from the copyright holder.
To view a copy of this license, visit https://creativecommons.org/licenses/by/4.0/

Referee #1 (Remarks to the Author):

This tour-de-force paper describes the operation of a sparse spin-qubit array in silicon. Using spin shuttling, the authors demonstrate weight-four parity checks and the generation of a 5-qubit GHZ state. The device design, tune-up, and operation are novel, and the demonstration of parity checks in a semiconductor spin-qubit platform is a major step forward. The experiments are very well done and clearly described, and the paper likely suitable for Nature. Here are my questions:

We thank the referee for their remarks about the impact of our manuscript and are glad they appreciate the novelty in the design, tune-up, and operation of the device.

1. Looking ahead, what are the main improvements, if any, that would need to be made to demonstrate elementary QEC with this platform? In addition to the state preparation fidelity, I also wonder about the time it takes to implement the parity check, how this compares with the expected coherence time of the physical qubits, and to what extent this matters for fault-tolerant QEC.

We have extended the discussion to highlight the concrete improvements and continued steps that would be required to demonstrate elementary QEC using the explicit example of the [[4,2,2]] error detecting code. We now highlight in the main text (with further details in Supplementary Note 3 and Supplementary Figure 3) the 4.84 us time required for a weight-four parity check in the present experiment and extrapolate the expected time for a full syndrome measurement using experimentally realistic shuttling velocities. By increasing the shuttling speed alone to 50 m/s, each parity check duration could potentially decrease by about 1.r us, at which point the single-qubit gates would pose the next time bottleneck (they too, could likely be shortened through additional parallelization or stronger EDSR driving, with both requiring sending more power to the device). We believe that with modest improvements, syndrome extraction could therefore take place in about 8 us, and parallel measurement of both ancillas on the same time scale is feasible (we used 10 us in experiment but can measure in as little as about 5 us at the expense of needing some more regular calibrations). With dynamical decoupling pulses occurring in approximately 4 us intervals, the average physical data qubit coherence time of 334 us would allow for the completion of about 20 rounds of error detection.

In the present device, we already demonstrate that the low 2Q fidelities are the most urgent performance bottleneck, and we comment on these below in response to point 2. As we now emphasize in the conclusion, all of the primitive operations used in the device have previously been demonstrated with fidelities exceeding 99% with the same device physics. We therefore include a coarse estimate in Supplementary Note 3 to suggest that a complete syndrome measurement would have a success probability of about 84%, with most of the error coming from data qubit idling.

We believe the reformulation of the conclusion, as well as the additional details we now outline in Supplementary Note 3, would permit those interested in spin-shuttling architectures to understand the expected performance of QEC experiments taking place in this and similar architectures with realistic fidelities.

2. Are the relatively low 2Q fidelities consistent with the observed levels of electrical noise, or is there another cause? What is the most likely method for improvement? Can the authors speculate on why relatively low 2Q fidelities were observed here in contrast to the higher 2Q gate fidelities in earlier work?

By comparing the measured 2Q fidelities (90-95%) to the quality factors of their oscillations (~20), we find reasonable agreement, suggesting that the 2Q gates are indeed limited by incoherent noise in the exchange coupling. Quality factors in earlier works reporting high-fidelity exchange-based gates were in the range of 50-100, and in a recent preprint from HRL have been improved to about 1000 through methodical engineering of the gate stack and heterostructure.

We have a few hypotheses for the low fidelities observed in this particular device. First, our barrier gates, being in the second metallization layer, have relatively strong lever arms for controlling the exchange coupling (for J~J_0\exp(alpha*BB), where BB is the barrier gate voltage, we measure alphas of 0.04-0.1 mV^{-1}). In other spin qubit works demonstrating high-fidelity CZ gates, this value has ranged from 0.024 mV^{-1} [37] to 0.04 mV^{-1} [51]. This means that for equivalent electrical noise, our exchange couplings will fluctuate more and therefore lead to lower fidelity. Second, we use relatively little attenuation, only having 20 dB at 4K for each fast control line. While this is very helpful for prototyping due to the large pulse amplitudes available on-chip, it also allows more noise to propagate to the device. A combination of redesigning the barrier gates to be smaller, or in a higher layer, to reduce their lever arm, as well as adding more attenuation at a lower temperature stage, would be helpful to achieve higher fidelities. We have made these remarks explicit in our commentary on the 2Q gate fidelities in the main text, and further remark on the details in Supplementary Note 1 which describes the electrostatic simulation of the device.

3. Do the extracted check errors also agree with expectations? The relatively simple error analysis for the GHZ state generation agrees with the calculated fidelities, but does this also hold for the check itself?

We can make an estimate similar to those of the GHZ state fidelities. The main difference for the check errors is that the data qubits are in spin eigenstates for the duration of the decoupled CZ operation, and therefore robust to dephasing during most of the check. As now detailed in the manuscript, we estimate a weight-4 Z-check error of 70.5% and a weight-4 X-check error of 70.0%, which are very similar to the measured check accuracies of about 72% and 67%

respectively. We have added these estimates to the main text and the justification for the calculation to the Methods (Error budget for logical state preparation).

4. The distribution of values for both the check accuracy and entangled state fidelity seem to significantly exceed the reported uncertainties. Why is this the case? Is this related to the experiment or the analysis?

The uncertainties reported arise from the bootstrapping protocol we use to estimate the spread (reported as ±1-sigma) of reconstructed check accuracies and state fidelities given the statistical uncertainty in the measured observables and readout corrections for each set of tomographic data. The uncertainties do not capture the differences in operational fidelity across the device, and therefore for parity checks and GHZ states involving different sets of qubits, we do not expect accuracies and state fidelities to all fall within the same intervals of uncertainty.

We would expect that repeated collections of tomographic data, assuming constant device behaviour, would therefore fall within this estimated spread. However, there is an inevitable aspect of device drift which we mitigate by performing routine calibrations. The presence of such residual drift is visible, for example, in some GHZ state fidelities (Fig 4f) that are anomalously low. Subject to this residual drift, consecutive measurements may exhibit variation that differs from that owing to statistics alone. We do not have a sufficiently large collection of repeated tomographic experiments to estimate such an interval of uncertainty. We have added a clear remark to this effect when discussing the maximum likelihood estimation procedure in the Methods (Quantum state tomography).

5. Can the authors briefly describe the full tune-up process, from the beginning?

We have added an additional Methods section (Initial device testing and tune-up procedure) and two figures (Supplementary Figures 4 and 5) that detail the I-V turn-on characteristics originally measured during device screening. We discuss how these measurements are used to provide an initial DC setpoint for the device tune-up. We then detail the order in which device operations are subsequently calibrated and refer to the respective Methods where appropriate.

Referee #2 (Remarks to the Author):

Undseth et al. present an impressive set of results showcasing the utility of coherent spin shuttling in increasingly complex semiconductor quantum devices. The manuscript goes well beyond prior work that only characterized the coherence of the shuttling protocol or used it as a local probe of valley splitting. The authors first describe a new device that incorporates readout, spin conveyor, and qubit zones. Figure 2 nicely showcases the capabilities of spin shuttling - using just a single charge detector at the left end of the device, the authors are able to remotely

tune-up qubits located as "bus stops" 1 - 4. The full loading, initialization, control and readout sequence is detailed in Fig. 3. Finally, the authors demonstrate weight-4 parity checks and the synthesis of a 5 qubit GHZ state in Fig. 4. The research is essentially moving the spin qubit community towards a significant quantum error correction demonstration, e.g. the [[4,1,2]] code.

Overall, the manuscript is of the highest quality and is most suitable for publication in Nature. On the whole, this research and other upcoming research by HRL as well as Diraq/imec are poised to bring spin qubits neck-and-neck with superconducting qubits. I therefore believe this is an important result that will reshape the perception of spin qubits in the broader community of quantum computing researchers.

We thank the referee for appreciating the quality of the manuscript and agree that this work, along with others including the recent preprint posted by HRL, highlights the progressing maturity of the spin qubit platform in the landscape of quantum hardware.

My only critical comment relates to performance metrics and projections for future work. While the work is impressive, the parity check accuracy of 67-73% is far below the commonly cited 99% threshold for the surface code. Two qubit gate fidelities are vastly limiting the overall performance of the device (up to 10% error, much worse than the best demos >99%). I therefore encourage the authors to provide a more balanced outlook for future work - many performance improvements are required before the basic ingredients contained in this demonstration can be put together to demonstrate a logical qubit. The final two paragraphs could be better put to use explaining how the remaining technological hurdles may be overcome.

We have followed the recommendation of the referee to provide additional details regarding the low 2Q fidelities (as detailed in response to Referee #1, point 2) as well as the subsequent steps required to realize near-term QEC experiments using this spin-shuttling architecture (as detailed in response to Referee #1, point 1). The final two paragraphs have been reformulated to provide a more balanced outlook. In addition to improving the 2Q gate fidelities, we highlight the remaining tune-up that would be required for the implementation of a [[4,2,2]] error detecting code, including optimizing the measurement time for mid-circuit measurements and tuning the right side of the device to operate as a second readout zone and mobile ancilla. As detailed in Supplementary Note 3, we conclude that conducting about 20 rounds of error detection should be possible, and that all necessary ingredients for achieving such performance have been demonstrated with Loss-DiVincenzo qubits well in excess of 99% fidelities. We believe the modifications make a stronger ending to the paper by making the path towards logical Loss-DiVincenzo spin qubits in shuttling architectures more tangible.

Referee #3 (Remarks to the Author):

In “Weight-four parity checks with silicon spin qubits” the authors introduce a new Si/SiGe spin qubit device and architecture and lay ground work for future quantum error correction (QEC) demonstrations by demonstrating spin shuttling, quantum non-demolition spin measurements, parity-checks, and GHZ states of up to five-qubits.

This paper is a tour de force of gate-defined spin qubit design, tune-up, and operation that pushes forward the state of the art of that field in its first appearance (I think it’s the first) and deserves a high-visibility publication somewhere. In my opinion, the significance of this work is less the weight-four parity checks highlighted in the title than the new device architecture they demonstrate. Unfortunately, the gate fidelities are not high, falling quite short of what’s necessary for even break-even error correction — and so I would dial back on the word “immediate” in the statement “This work opens immediate opportunities to pursue QEC experiments with spin qubits.” The quantum information concepts here are well-explored and better performant in other quantum technologies and it’s less obvious to me what this work offers to those outside of the gate-defined spin qubit community. I would defer to other referees more further afield as to whether this work is of sufficient broad interest for Nature as opposed to one of its more specialized journals.

We thank the referee for their appraisal of our manuscript and appreciation of the significance of the architecture. Considering the reviewer’s comments as well as other feedback we have received from the community, we have opted to amend the title to “Weight-four parity checks in a spin-shuttling architecture”. We believe this places a more balanced emphasis on not only the parity checks, but also on the advances in control techniques used to achieve them. We have also followed the suggestion of the referee and removed the word “immediate” from the abstract. Instead, we reformulate the final sentence to emphasize how the parity checks demonstrated here may be used in near-term QEC experiments.

This device is the most ambitious shuttling-based spin qubit device that I’m aware of, which is the source of much of its power. As the authors highlight, shuttling adds flexibility to a spin qubit device design, facilitating effective long-range and reconfigurable interactions between data qubit sites. It also permits a sparser layout, which can be strategically employed to mitigate routing density and cross-talk challenges with this technology. Qubit shuttling is not unique to gate-defined spin qubits, and is a core characteristic of other quantum platforms, but perhaps this is the most capable demonstration in a solid-state platform. Specifically in this demonstration, the authors use the shuttling to realize a demonstration of a surface-code stabilizer plaquette and to do this on an extended device (9-dots-deep, in a sense) with only a single readout site.

The tune-up of this device with such a remote, single readout site is remarkable, with a number of innovations detailed in the main article and supplement. These descriptions take up much, maybe most, of the article, but are probably of less interest to those outside the field, I fear.

My biggest concern about the viability of the design — even in the immediate term — are the relatively low 2-qubit errors achieved. The authors point out that several similar devices have achieved 2-qubit errors significantly below 1% error, but all such errors in this device are between 10% and 5%, without explanation, suggesting some systemic issue in the design, processing, or control. What are some of the possibly relevant differences between this work and those other, higher performant ones? What evidence is there for a straightforward path for ab improvement of 10x or more without a significant change to the design or approach? For example — to simply pick a hypothetical trade to illustrate my general concern — if a lot of the issue is a too-small lever arm of the BB(X) gates because it's higher in the stack, solving this issue by reordering the stack or changing the gate shape would likely trade with shuttling fidelity, or maybe some other figure of merit.

We now provide additional details regarding the low 2Q fidelities in both the Methods as well as the Supplementary Information (as detailed in response to Referee #1, point 2) which include concrete comparisons to previous high-fidelity 2Q gate demonstrations in similar devices. We speculate that the modest device design and setup improvements necessary to achieve >99% fidelity 2Q gates will not lead to a fundamental trade-off with other device performance, such as shuttling, owing to our ability to virtualize control of the array. It may, however, require more care in calibration. For example, in the present experiment, the very large dynamic range of exchange control made uncoupling the data qubits from the shuttled spin very easy. If an improved design for higher 2Q fidelities with smaller barrier lever arms is realized, the DC voltage would need to be more carefully chosen to allow for adequate exchange control.

Similarly, while the shuttling fidelity is good — and the authors claim it's the highest performant by some metrics — incurring a >2% error moving the ancilla around also needs to be improved at least 10x for error correction viability. The authors suggest vaguely that shuttling faster would improve the fidelity, but going faster always exacerbates other issues like control fidelity or state transitions. My general point is that although the authors demonstrate the functional ability of some key QEC operations, there's not much evidence that the performance issues have a clear path to improvement without some unknown larger changes, and so even the short term viability of the design has questions.

We agree that the shuttling fidelity would also need to improve to reach favourable QEC territory. In short, because of the much larger bottleneck of 2Q fidelities, there were diminishing returns to maximizing the performance of other primitives, such as shuttling and readout. We cannot, at present, prove that a 10x increase in shuttling fidelity is possible in this particular sample, but we can point to the much higher fidelity and speed achieved in [14] as proving the feasibility (including the possible benefit of using an 8-phase conveyor as opposed to the 4-phase conveyor employed here). We can add that further optimization in the same device as used in this manuscript has already improved the round-trip shuttling fidelity to 98.5%.

Associated smaller questions and feedback:
Fig 1a: A silly aesthetic nit-pick, but the four “bus stops” are depicted as “buses,” while the shuttling electron is really the moving vehicle. Sorry, this might just be me. I might be the only person who would think about this.

We have used the standard indication of a bus stop in the Netherlands. While we appreciate the aesthetic critique, we are inclined to keep the present depiction for its simplicity as opposed to adding the text “Bus stop” that may clutter the figure.

Fig 1c: The legend doesn’t clearly specify the circles as either “simulation” or “measurement.” It can be deduced by some context that they are measurements, but there’s probably a clearer way to organize the legend.

We have amended the legend by also indicating a circle next to the “Measurement” label to clarify that the circular qubit markers also refer to measurements.

Page 3: there is a comment that the slowest operation is PSB readout, which takes 10 us. Given that this long integration likely comes at other fidelity costs or complications, it might be illuminating to explain more in the supplement how this integration time was chosen.

We now emphasize in Methods (Ancilla readout) that 10 us was chosen as a default integration time such that decent readout fidelity could be maintained without requiring recalibration more than about once per day. We have pushed the integration time to as short as 5 us while maintaining similar visibilities, but more frequent calibration of the single-shot threshold signal is required to maintain performance. The original 10 us time was selected based on past experience but not optimized more carefully. In Supplementary Note 3 detailing a near-term [[4,2,2]] implementation, we highlight how in future experiments leveraging mid-circuit measurements, a more systematic balancing of readout time and fidelity would be required.

Fig 3g: Why is the ancilla 1Q gate 10x worse than the data qubit 1Q gates?

We cannot give a definitive reason for this, but we speculate that it has to do with the proximity of the spin to the reservoir. We have added this comment in the Methods (Single-qubit characterization). Based on our experience, it appears that more isolated spins, such as those localized in the bus stops in our device, generally exhibit higher fidelities.

Page 5: There is a comment that the ancilla is shuttled back from the D4 qubit to P2 to perform a higher fidelity 1Q decoupling pulse, but this would seem unlikely given the 2.3% fidelity hit this

extra shuttling would incur? Do the ancilla 1Q gates not under P2 have 3% or worse error? Or am I misunderstanding something?

We have added a paragraph to the Methods (Single-qubit characterization) to elaborate why we made this choice. The biggest reason is experimental convenience. Although the choice of shuttling back is redundant, wastes idling time and incurs a fidelity hit, it is easier to apply all single-qubit gates when the device voltages are at the same set point (e.g. in our case when the conveyor potential is off). Otherwise, small resonance frequency shifts need to be accounted for, and the calibration overhead increases. There are other reasons we chose to avoid this in the present demonstration. The addressability between the ancilla and D4 is not very large when the two are adjacent, so extra care of synchronizing control would have to be taken. Finally, although EDSR control is possible throughout the conveyor trajectory, it is not necessarily high fidelity. All of these challenges could be overcome with some effort, and we believe there would be merit to only controlling A1 when in the conveyor potential, since much of the shuttling error is incurred when loading the spin from below P2 to below C0.

Page 6: The authors claim that this is the first observation of "genuine five-qubit entanglement" in a gate defined semiconductor spin, and while this may be true, the observed fidelity appears to be ~53%, which is quite marginal, especially considering the probability corrections in state tomography (no matter how common and an accepted, necessary evil such corrections may be). This claim cannot be given much weight, unfortunately.

We agree that the 53% is not remarkably high, but we were inclined to highlight this value both because it is the largest GHZ state demonstrated with gate-defined semiconductor spin qubits, and it is achieved despite the modest 2Q gate fidelities and the "penalty" incurred by shuttling. In truth, we expect the actual 5Q state fidelity to be closer to the 4Q GHZ fidelity (~63%) that is prepared via a parity check, as this procedure requires a 5Q entangled state. As we now emphasize in the Methods (Error budget for logical state preparation), these circuits are effectively identical, but the 5Q tomography takes about 2 hours, over which we believe parameter drift begins to play a substantial role in limiting the fidelities.

Page 7: The authors claim this approach could tune up 100 qubits with a single charge sensor? What sets this limit? (e.g. Why not 1000?). What shuttling fidelity is this assuming, and why is this plausible?

We have added our reasoning to the Methods (Single-spin remote tuning) to justify the estimate. We use the trilinear architectures currently manufactured by imec, HRL, and Intel as a concrete example, and extrapolate how many bus stops would effectively fit in such a device if the central row was a shuttling channel of reasonable fidelity. We use 10 microns as an example, as shuttling over this effective distance has already been established, and estimate that about 110 bus stops with our dimensions could exist in such a device, assuming the charge sensor is located

at one end of the array. As our remote tuning protocol requires only some spin coherence to infer charge stability diagrams, there is not a strict requirement for high-fidelity spin shuttling. While such an estimate requires speculation about the performance of shuttling over much longer length scales, we believe proposing a plausible number is superior to simply stating our method is “scalable”. We now also highlight in the main text that the purpose of such multiplexing would be to allow charge sensors, which are relatively bulky compared to spin qubits, to be allocated according to their need for quantum information processing rather than being necessary for device tune-up.

Appendix G: What are the T1(0,1) times?

Given the dynamic loading protocol, we are unable to wait sufficient time to observe T1 decay in the bus stops as we are limited to waiting about 20 ms by the bias-T times of the sample board. We have also not measured the T1 times of the readout pair. From measurements with similar devices, we would expect the T1 time to be on the order of 100s of milliseconds. We now state this explicitly in the Methods (QND measurement).
